# Metastatic large cell neuroendocrine lung cancer to the foramen magnum: A case report

**DOI:** 10.1097/MD.0000000000021628

**Published:** 2020-08-14

**Authors:** Peng Zou, Jian Liu, Guang Cheng, Kai Wang, Ao Li, Sanzhong Li, Yangang Wang, Xifeng Zou, Xituan Ji, Dakuan Gao, Weiping Liu, Xiaofan Jiang

**Affiliations:** Department of Neurosurgery, Xijing Hospital, Air Force Medical University, Xi ’an Shaanxi, China.

**Keywords:** large cell neuroendocrine lung cancer, the foramen magnum

## Abstract

**Rationable:**

Large cell neuroendocrine carcinoma of the lung is rare, especially in the area of the foramen magnum. No previous studies have reported metastatic large cell neuroendocrine lung cancer to the foramen magnum. This paper will be the first time to report this special case.

**Patient concerns:**

A case of a 37-year-old woman presented with headache that had developed 20 days previously. Imaging examination revealed a circular abnormal signal at the posterior margin of the foramen magnum.

**Diagnoses:**

The patient we report was diagnosed with a metastatic intracranial tumor.

**Interventions:**

The patient underwent occipital craniotomy. Pathological results showed metastatic neuroendocrine carcinoma of the brain. Whole body PET-CT examination showed that fusiform soft tissue shadows could be seen near the hilum of the lower lobe of the left lung.

**Outcomes:**

The final bronchoscopy pathological results showed the large cell neuroendocrine carcinoma of the lung. The patient underwent further chemotherapy and radiotherapy in the oncology department.

**Lessons:**

Diagnosis and treatment of large cell neuroendocrine carcinoma of the lung are difficult. The prognosis is poorer, and effective treatment is urgently needed.

## 1 Introduction

Neuroendocrine tumors are epithelial neoplasms with predominant neuroendocrine differentiation and, while typically seen of pulmonary origin, can arise in most organs.^[1]^ The 2004 World Health Organization (WHO) classification proposed four subtypes of pulmonary neuroendocrine (NE) tumors: low-grade typical carcinoid (TC), intermediate-grade atypical carcinoid (AC) and two high-grade tumors, large cell neuroendocrine carcinoma (LCNEC) and small-cell lung carcinoma (SCLC). LCNECs of the lung represent a rare subtype of primary lung cancer accounting for only 2.9% of cases. Tumors of the foramen magnum are rare, accounting for 0.6% of primary intracranial lesions.^[2]^ Meningiomas, schwannomas, and ependymomas are the most frequent tumors and others include chordomas and epidermoid cysts. However, metastatic neoplasms located in the foramen magnum are extremely uncommon.^[3]^ Brain metastases are the most commonly found at the junction of the hemispheric gray and white matter. To the best of our knowledge, no studies have reported metastatic large cell NE lung cancer to the foramen magnum.^[4]^ This paper will be the first time to report this special case. Here, we report a female patient who was diagnosed with NE carcinoma of the lung with metastasis to the foramen magnum. This research was approved by the Ethics Committee of Xijing Hospital Affiliated to Air Force Military Medical University, and informed written consent was obtained from the patient for the publication of this case report and accompanying images.

## 2 Case report

**Figure 1:**
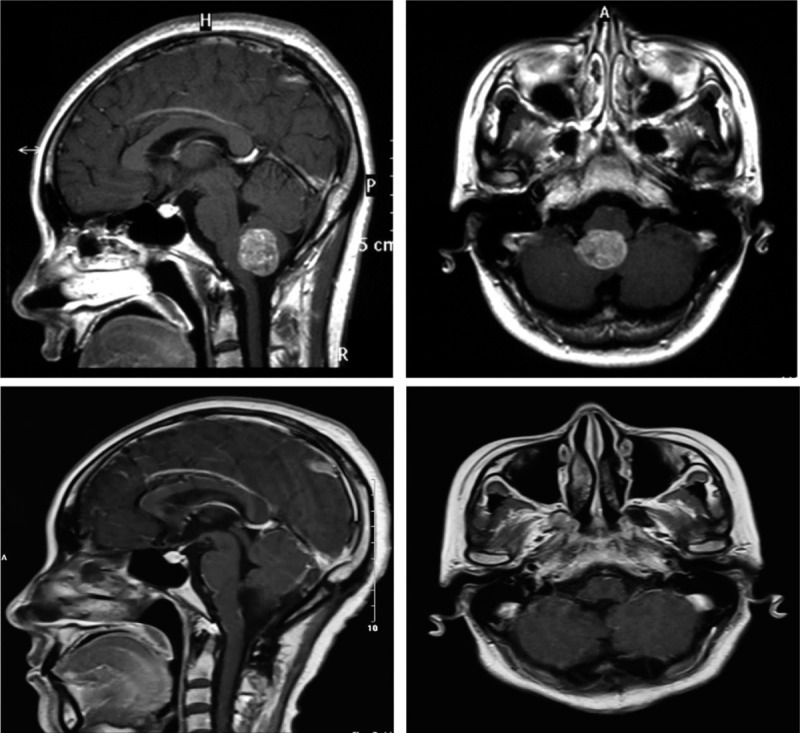
Preoperative cranial magnetic resonance suggested a circular abnormal signal at the posterior margin of the foramen magnum, with clear boundaries and a size of about 2.5 cm × 1.9 cm × 2.6 cm. After enhanced scanning, the signal was significantly enhanced, and the dorsal medulla oblongata was compressed. Postoperative MRI on the second day after surgery suggested complete resection of the tumor.

**Figure 2:**
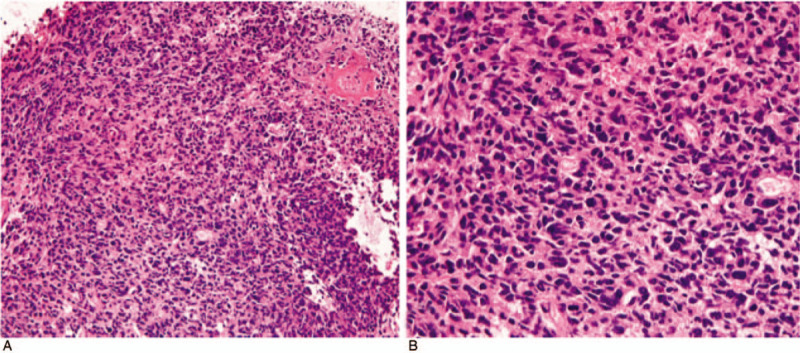
The histological features of brain tumors were as follows: nest arrangement of heterosexual cells, large and dark nuclei, high cell density, and scattered mitosis. HE = A × 50; B × 200.

**Figure 3:**
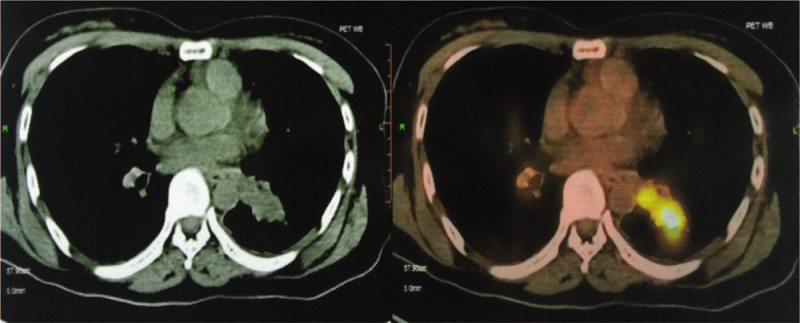
Whole body PET-CT showed that fusiform soft tissue shadows with a size of about 43 mm × 22 mm × 32 mm could be seen near the hilum of the lower lobe of the left lung. Glucose metabolism was increased and the edges were lobulated. Malignant lesions were mainly considered.

A 37-year-old female presented to the emergency department complaining of headache for 20 days. Major symptoms were paroxysmal pain and irregular onset, accompanied by nausea and vomiting. Physical examination showed no positive signs except for a positive Romberg sign. Laboratory tests were negative. Cranial enhanced MRI showed there was a circular abnormal signal at the posterior margin of the foramen magnum, with clear boundary and a size of about 2.5 cm × 1.9 cm × 2.6 cm. After the enhanced scan, the signal was significantly enhanced. This mass compressed the dorsal medulla oblongata and was closely related to the spinal cord. The supplying artery was the posterior inferior cerebellar artery. We suspected a schwannoma or ependymoma. In order to improve the patient's clinical symptoms and obtain an accurate diagnosis, the patient underwent occipital craniotomy through the posterior median approach. During the operation, the posterior occipital bone and C1 posterior arch were opened, and the dural membrane was incised in a y-shaped manner. The tumor was found to be located below the cerebellar tonsils at the posterior margin of the foramen magnum. It was closely related to the right posterior inferior cerebellar artery, with clear boundary and soft texture. The tumor was removed completely and sent for pathological examination. After the operation, the patient's vital signs were stable. Consciousness was clear and no abnormal signs were found. Cranial enhanced MRI re-examination on the second day after surgery showed complete resection of the tumor (Fig. 1). Pathological results showed metastatic NE carcinoma of the brain, the Ki67 value-added index was as high as 95%. Immunohistochemistry showed that AE1/AE3, CD56, CK8/18, EMA, Syn, and TTf-1 were positive, while CD34, CgA, CK7, GFAP, Nestin, NeuN, S-100, and Vim were negative (Fig. 2). Whole body PET-CT examination showed that fusiform soft tissue shadows with a size of about 43 mm × 22 mm × 32 mm could be seen near the hilum of the lower lobe of the left lung (Fig. 3). After consultation with the department of respiratory and oncology, Bronchoscopic lung biopsy was performed and the final pathological results indicated the LCNEC of the lung. The patient was diagnosed as stage IV large cell lung NE malignancy. Subsequently, patient received prophylactic radiotherapy and three cycles of cisplatin-etoposide. Following the chemotherapy regimen, and considering his poor condition, the patient was provided only with supportive care. He died 5 months after receiving the diagnosis of LCNEC.

## 3 Discussion

Large cell neuroendocrine carcinoma is part of lung NE tumors, accounting roughly for 2.9% of all lung malignancies.^[5]^ LCNEC, introduced in 1991 by Travis et al, represents an extremely rare entity with aggressive behavior and poor prognosis.^[6]^

The case we report has two main characteristics. One is that the large cell neuroendocrine tumor of the lung is extremely rare. The other is that the site of metastasis to the foramen magnum is extremely rare. We searched PubMed using the term “neuroendocrine lung cancer”, and 12 English reports of clinical cases were reviewed^[1–12]^ (Table 1). Among these cases, there were 7 female patients and 5 male patients. The average age was 50.21 ± 6.96 years. Seven patients’ tumors had metastasized to the head. Other sites of metastasis are stomach, liver, breast, esophagus, and cervix. Among the 7 cases of intracranial metastasis, 2 in frontal lobe, 2 in temporal lobe, 2 in parietal lobe, and 1 in pineal region. No reports of metastatic large cell NE lung cancer to the foramen magnum were found. The present report likely represents the first case.

**Table 1:**
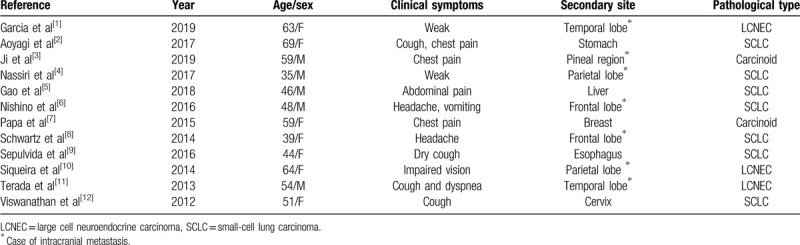
Literature review of neuroendocrine lung cancer.

Tumors of the foramen magnum are rare, accounting for 0.6% of primary intracranial lesions.^[2]^ Meningiomas, schwannomas, and ependymoma are the most frequent tumors and others include chordomas and epidermoid cysts.^[3]^ Metastatic neoplasms located in the foramen magnum are extremely uncommon, because brain metastases are the most commonly found at the junction of the hemispheric gray and white matter. It is reported in the literature that the tumors metastasized to the foramen magnum can come from prostate adenocarcinoma and malignant melanoma, but the tumors from NE cancer have not been reported.^[4]^ So far, few brain metastatic NE tumors have been reported worldwide, as well as lack clinical data and relevant treatment guidelines, therefore it brings great difficulties and challenges to our diagnosis and treatment of this patient.

Diagnosis and treatment of LCNEC is difficult. Smoking is an independent risk factor for the disease, but the case we report has no smoking history. Surgery and immunohistochemical staining can confirm the diagnosis. Primary surgery is the mainstream of treatment, although it is rarely amenable due to local or systemic tumor metastasis at the time of the diagnosis. Neuroendocrine markers such as somatostatin, synaptophysin, and pheochromogranin were examined to confirm this diagnosis.^[7]^ According to the latest literature report and experts, current recommendations are based on the expansion of chemotherapy regimens for small cell lung cancer, with 4 to 6 cycles of etoposide plus cisplatin chemotherapy recommended.^[8]^ In 2016, Lyoda et al used etoposide plus cisplatin chemotherapy in a prospective clinical study diagnosed as LCNEC, and the results confirmed that the chemotherapy group was superior to the control group.^[9]^

Because LCNEC is rare, the optimal treatment has not been defined. Based on the National Comprehensive Cancer Network (NCCN), treatment should be based on NSCLC recommendations. Usually, the most used schedule is platinum-based chemotherapy, however, the outcome remains poor. Recent studies showed that LCNEC responds to cisplatin-based chemotherapy, like those used for SCLC.^[6]^ Gamma knife treatment may be a favorable approach. Prophylactic cranial irradiation has shown a clear role in the survival benefit in LCNEC.^[8]^ Surgery should be offered in all operable patients. In this case, we consider that the lesion is single and compresses the brainstem. If not operated, it may be life-threatening or hydrocephalus. Hence, we perform craniotomy.

Within the lung malignancies with non-small histologies, LCNEC is cancer with a worse prognosis, even in early stages after complete resection.^[10]^ Dresler et al demonstrated a 5-year survival for stage I LCNEC cases of 18%. In contrast, based on Iyoda et al's results, this disease encompasses a 5-year survival rate of 35.5% and a 5 year disease-free survival rate of 27.4%.^[11]^ Despite a multimodal approach, previous retrospective studies have reported poor survival outcomes.^[12]^ The clinical presentation might be similar to other types of lung cancer, however, histological diagnosis is needed in order to direct more specific therapy.

## Author contributions

**Conceptualization:** Peng Zou.

**Data curation:** Peng Zou.

**Formal analysis:** Peng Zou.

**Funding acquisition:** Peng Zou.

**Investigation:** Peng Zou.

**Methodology:** Peng Zou.

**Project administration:** Peng Zou.

**Resources:** Peng Zou.

**Software:** Peng Zou.

**Supervision:** Peng Zou.

**Validation:** Peng Zou.

**Visualization:** Peng Zou.

**Writing – original draft:** Peng Zou.

**Writing – review & editing:** Peng Zou.
